# Optical Devices Constructed From Responsive Microgels for Polyphenols Detection

**DOI:** 10.3389/fchem.2021.580025

**Published:** 2021-03-11

**Authors:** Jingying Wang, Xieli Zhang, Kaiyao Shi, Qiang Zhang

**Affiliations:** ^1^ Department of Laboratory, 15189 Accredited Laboratory, Jilin Province Drug Resistance Monitoring Center, China-Japan Union Hospital of Jilin University, Changchun, China; ^2^ State Key Laboratory of Electroanalytical Chemistry, Changchun Institute of Applied Chemistry, Chinese Academy of Sciences, Changchun, China; ^3^ School of Applied Chemistry and Engineering University of Science and Technology of China, Hefei, China; ^4^ Provincial Key Laboratory for Gene Diagnosis of Cardiovascular Disease, Jilin Provincial Engineering Laboratory for Endothelial Function and Genetic Diagnosis, Department of Cardiology, China-Japan Union Hospital of Jilin University, Changchun, China

**Keywords:** microgels, optical devices, polyphenols detection, responsive polymer, photonic crystal (PC)

## Abstract

Polyphenols are used as antioxidants in various foods and beverages, which are considered to be a health benefit. The measurement of polyphenols contents is of great interest in food chemistry and health science. This work reported a microgels based photonic device (etalon) to detect polyphenols. Dopamine was used as a model compound of polyphenols. Herein, we proposed a “block” concept for dopamine detection. The dopamine was oxidized and formed dopamine films catalyzed by tyrosinase on the surface of etalon. As the etalon was immersed in ZnCl_2_, the dopamine films blocked the ZnCl_2_ diffusion into etalon that caused optical property changes. The film thickness is associated with the concentration of dopamine which can be readout via optical signals.

## Introduction

Polyphenols are strong antioxidants existing in fruits and beverages like tea, red wine, and coffee, which play an important role in human health (Ponder and Hallmann, 2019; Zheng et al., 2020). The catechol structure allows polyphenols to scavenge free radicals and inhibit lipoprotein oxidation, reducing the incidence of cardiovascular diseases and some cancers (Ru et al., 2019; Gao et al., 2020; Zhang et al., 2020). Therefore, it is very important to detect such compounds in foods in the fields of food chemistry and healthy science. Up to now, some methods have been developed for polyphenols detection, such as chemiluminescence (Nalewaiko-Sieliwoniuk et al., 2015; Nalewajko-Sieliwoniuk et al., 2016; Zhao et al., 2017), mass spectra (Fayeulle et al., 2019), electrophoresis (Yasui and Ishii, 2014; Matei et al., 2016; Parveen et al., 2016), electroanalysis (Fu et al., 2012; Thangaraj et al., 2012; Alcalde et al., 2019) and chromatography (Hashim et al., 2020; Ovchinnikov et al., 2020; Yuan et al., 2020). However, these methods get involved with complicated sample purification, and the complex composition and the presence of interferences in the extracted samples often caused big experimental errors during polyphenols content measurement. Meanwhile, these detection are expensive and require professional technicians to complete, which significantly restricts their abroad applications.

Photonic materials are composed of close packed arrays of nanoparticles that self-assemble into crystal structures. As light illuminated the crystal structure, it will be constructed or deconstructed in a certain direction to generate an interference light. The color and optical properties (e.g., reflected light wavelength) of photonic materials depends on the distance between lattice planes. In recent years, photonic materials have been explored as colorimetric sensors due to their attractive features such as visual readout, low cost, and easy handling (Hou et al., 2018; Zhang et al., 2018). For example, the Asher group developed a series of two-dimensional photonic crystals that were prepared by locking 3D ordered colloidal particles in hydrogels (Cai et al., 2015; Cai et al., 2016). Then, they utilized these materials to sense various chemical small molecules and biomacromolecules (Zhang et al., 2011; Zhang et al., 2013; Zhang et al., 2014b). Similarly, 3D photonic crystals have also been reported and used to sense various environmental factors, such as metal ions (Liu et al., 2020), RNA (Li et al., 2020), gas (Huang et al., 2019; Afsari and Sarraf, 2020; Huang et al., 2020), protein (Cai et al., 2016; Cai et al., 2017), and bacteria (Massad-Ivanir et al., 2014; Hou et al., 2018; Zhang et al., 2018). Our group prepared specific microgel-based optical devices (i.e., an etalon). We subsequently studied their optical properties and utilized the device to sense various environmental analytes, e.g., triglycerides (Zhang et al., 2015a), Tabun (Zhang et al., 2014b), Cu^2+^ (Zhang et al., 2015b), CO_2_ (Zhang et al., 2015c) and H_2_O_2_ (Zhang et al., 2016). Briefly, etalons were prepared by depositing a single layer of microgels on an Au-coated glass substrate, and then another layer (15 nm) of Au was deposited on the microgels to form a sandwiching structure (Zhang et al., 2014a). The optical properties of etalon can be described using Eq. 1:

mλ=2ndcosθ (1)

where *n* is the refractive index of the microgels layer, d is the space between two Au layers, *θ* is the angle of incident light relative to the normal, and m is the order of reflected peaks that should be integers. Therefore, the reflection wavelength and etalon colors are dependent on the distance of Au-Au layers and the refractive index of microgels.

In the submission, we developed a “block” concept for polyphenols determination using etalons. First, the surfaces of etalons were modified using tyrosinase that could catalyze polyphenol oxidation generating polyphenol films on the etalon surface in presence of O_2_. The thickness of polyphenol films depends on the concentration of polyphenols. When the device was soaked in ZnCl_2_ solution, the polyphenol films blocked Zn^2+^ entering into etalon that caused optical property changes of etalons. Therefore, the concentration of polyphenols can be deduced according to optical property change of etalons. In this paper, dopamine was used as a mode compound of polyphenols for polyphenol detection research. In a comparison of electrochemical methods, mass spectroscopy-based methods, and fluorescent probes, this system does not get involved with bulk and expensive equipment, complicated sample pre-treatment, and complex operation.

## Experimental Section

### Synthesis of Microgels (MG-AAc)

N-isopropylacrylamide (11.9 mmol), acrylic acid (0.65 mmol), and N,N′-methylenebisacrylamide (0.65 mmol) were dissolved in 100 ml deionized (DI) water, and then the solution was filtered using a 0.45 μm filter. The solution was added into a 3-necked round bottom flask with a reflux condenser, nitrogen inlet, and temperature probe. The solution was heated to 70°C under N_2_ atmosphere. The polymerization was then initiated using ammonium persulfate (0.2 mmol) in 1 ml of DI water. After 4 h reaction, the resulting suspension was cooled to room temperature and the filtered using Whatman #1 filter paper to remove any large aggregates formed during the polymerization. The microgel solution was purified for six times via centrifugation to remove unreacted monomers and supernatant. The purified microgels solution was kept in a brown glass jar before use.

### Modification of Etalon Surfaces With Tyrosinase

The etalon was cleaned by rinsing with deionized (DI) water and ethanol separately. N-hydroxysuccinimide ester groups were attached on etalon surface by soaking into 3,3′-Dithiodipropionic acid di (N-hydroxysuccinimide ester) (DTSP) solution at a concentration of 0.1 mmol ml^−1^ for 2 h. Then, the etalon was taken out from solution, followed by washing with massive ethanol and stored in ethanol solution overnight to remove residual DTSP.

Tyrosinase was fixed on the etalon surface through covering the DTSP functionalized etalon with 100 μL tyrosinase aqueous solution (1 mg ml^−1^) for 2 h. The residual tyrosinase was removed by rinsing the etalon surface with massive DI water.

### Etalon Pre-Treatment Using Dopamine

100 μL dopamine aqueous solutions at a certain concentration were sprayed on the etalon surfaces. After 2 h, the dopamine aqueous solutions were removed from the etalon surfaces using micropipette, followed by washing with massive DI water to remove unreacted dopamine monomer from polydopamine film. The edge of these etalons was sealed using nail polish to prevent solvent and solute entering etalon from periphery.

### Reflectance Spectroscopy

Reflectance measurements were completed in a home-made sample holder using a USB2000 + spectrophotometer, a HL-2000-FHSA tungsten light source, and a R400-7-VISNIR optical fiber reflectance probe, all from Ocean Optics (Dunedin, FL). The spectra were recorded using Ocean Optics Spectra Suite Spectroscopy Software over a wavelength range of 350–1025 nm. Measurements were performed in the sample holder that leads to careful sample positioning, test stability, and precise temperature control.

## Results and Discussions

**FIGURE 1:**
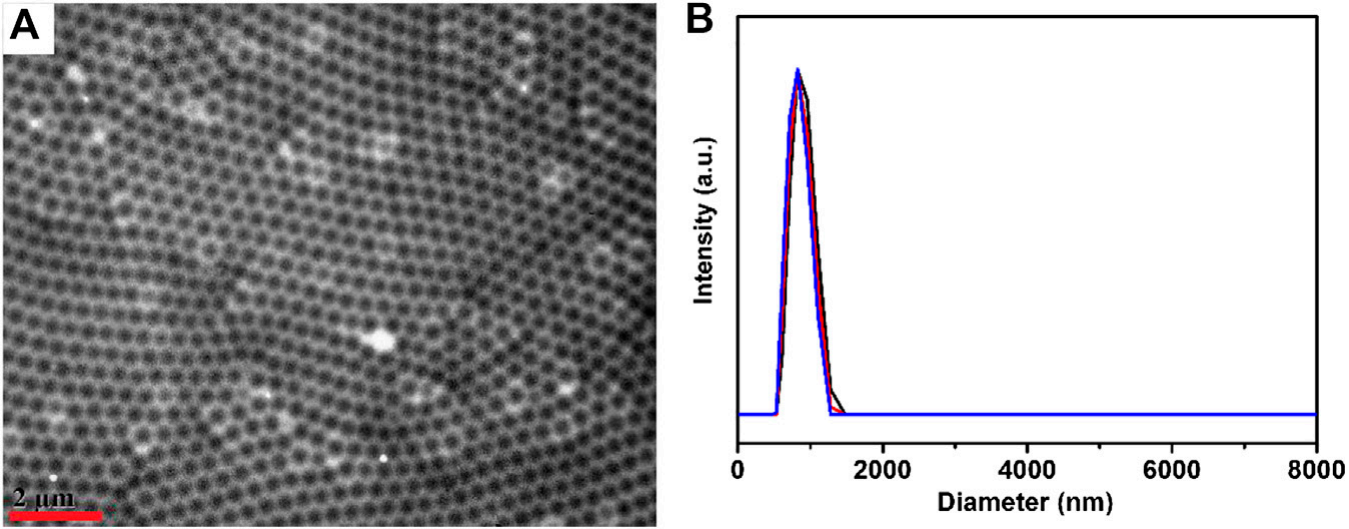
**(A)** TEM image of microgels; the scale bar stands for 2 μm **(B)** microgel diameters at 30°C determined three times by DLS.

**FIGURE 2:**
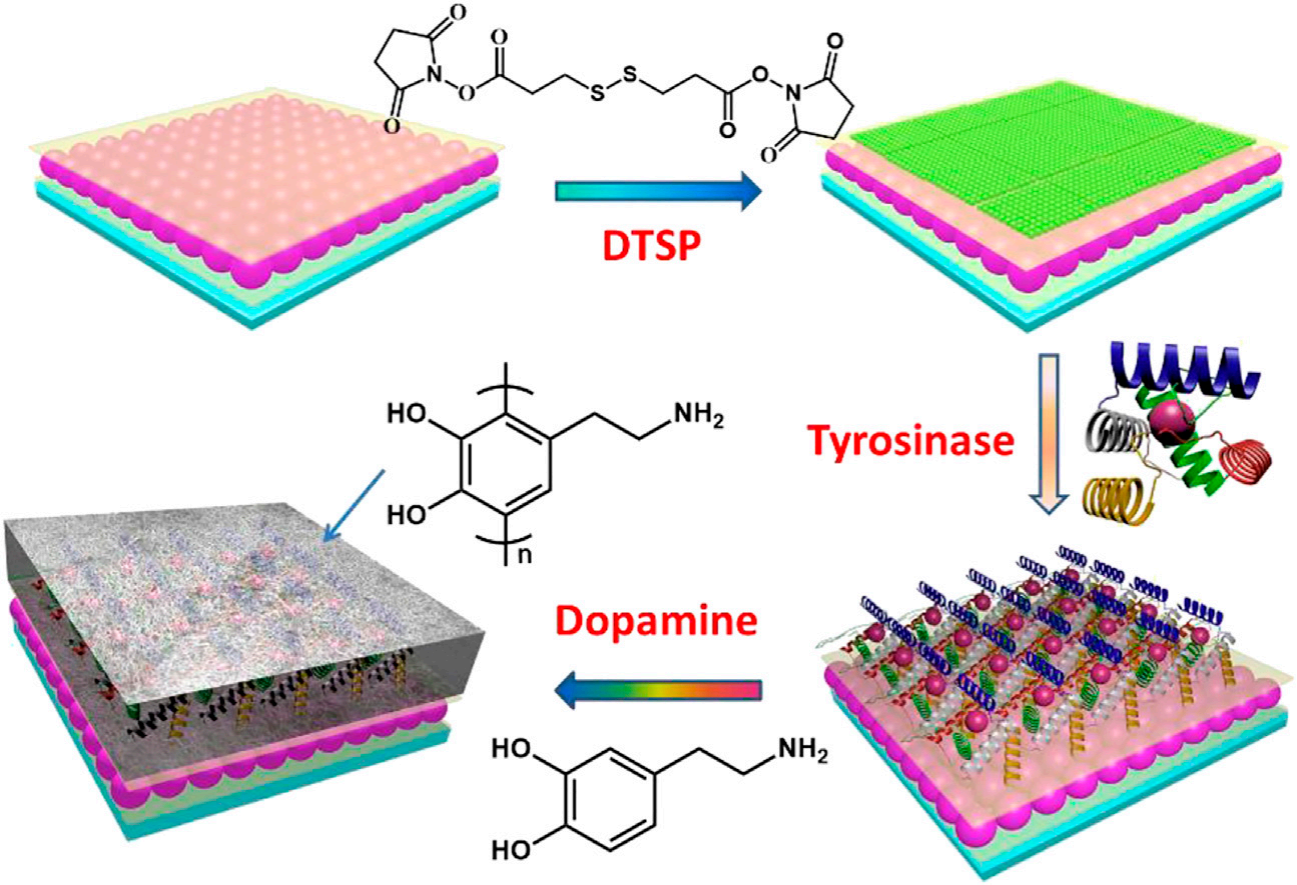
Schematic process of etalons.

First, microgels were synthesized by the precipitation polymerization of N-isopropylacrylamide, acrylic acid, N, N′-methylene bis (acrylamide) (crosslinker). The resultant microgels have been characterized using transmission electron microscope (TEM) and dynamic light scattering (DLS), as shown in Figure 1. The microgels exhibited the diameters of ∼340 nm (±10 nm) in dried state and diameters of 840 nm (±15 nm) in swelling state (polydispersity index: 1.03) at 30°C. The characterized microgels were then used to construct microgel-based etalons following previous reported protocol by our group (Xia et al., 2020). These etalons were comprised of single layer microgels sandwiched by two semi-transparent gold layers on glass substance, as shown in Figure 2. In order to deposit polyphenol films on the surfaces of etalons, we covalently fixed tyrosinase on etalon surfaces that can catalyze catechol oxidation generating benzoquinone and polyphenol films (Solem et al., 2016). Firstly, oxysuccinimide ester groups were attached on etalon surface via Au–S bonds by soaking etalons in DTSP solution (1 mg ml^−1^). Tyrosinase was subsequently fixed on etalon surfaces through substitution reaction between amine groups of tyrosinase and oxysuccinimide ester groups on etalon surfaces. When catechols present in the system, tyrosinase catalyzes oxidation reactions of catechol into o-quinone (Desentis-Mendoza et al., 2006). Subsequently, a conjugate addition of diverse nucleophiles (catechols) with o-quinone takes place (Espín et al., 2001). These reactions repeatedly happened yielding polymers with high molecular weights and further forming polymeric films. The solute was prevented from entering into the etalon from edges by sealing fringes with nail polish. The schematic process of etalons was shown in Figure 2.

**FIGURE 3:**
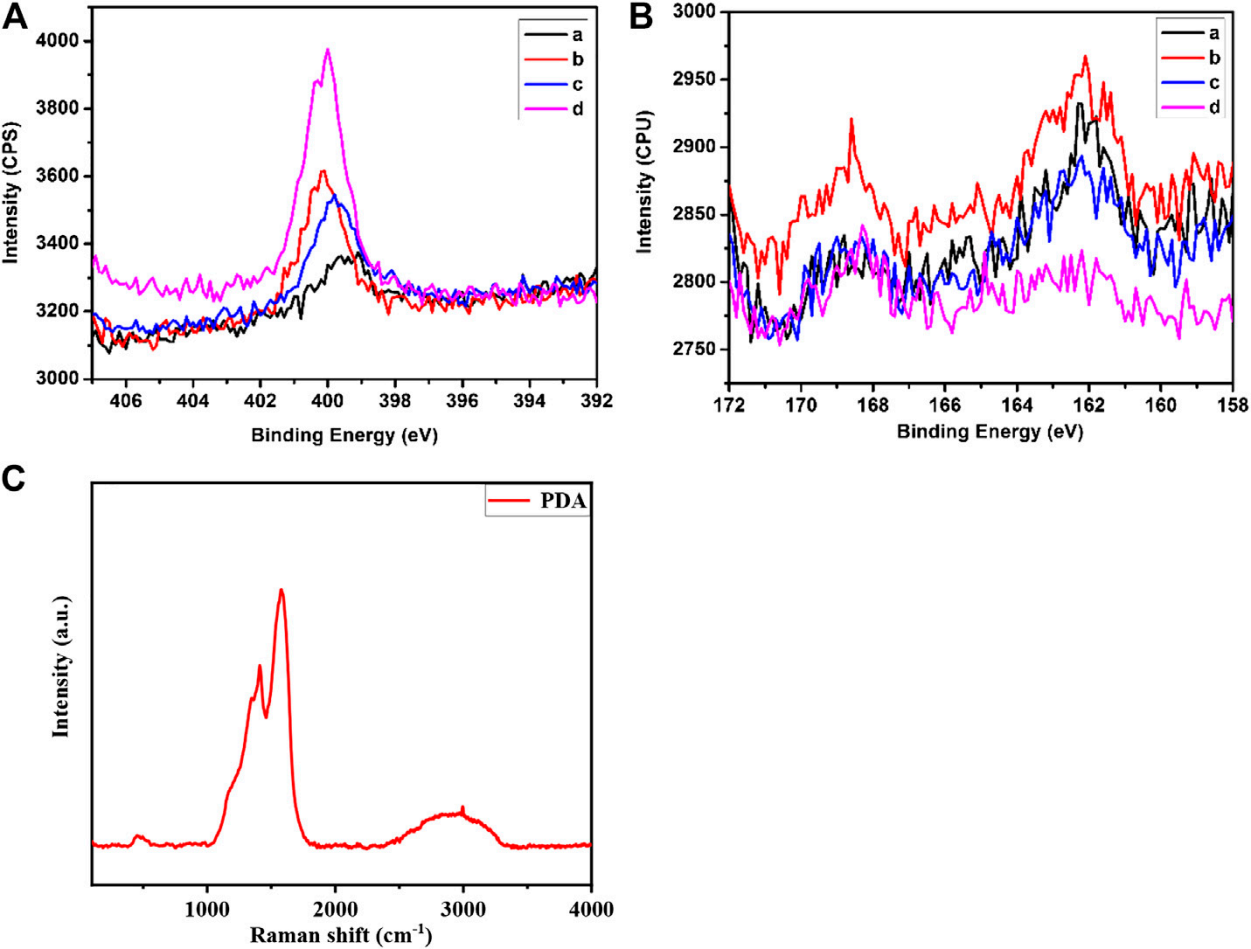
Element-specific high-resolution XP spectra of N1s **(A)** and S2p **(B)** regions. Panels in the two pictures: a: etalon; b: etalon modified with DTSP; c: etalon modified with tyrosinase; d: etalon modified with polydopamine film **(C)** The Raman spectra of polydopamine film on the surface of the sensors.

**FIGURE 4:**
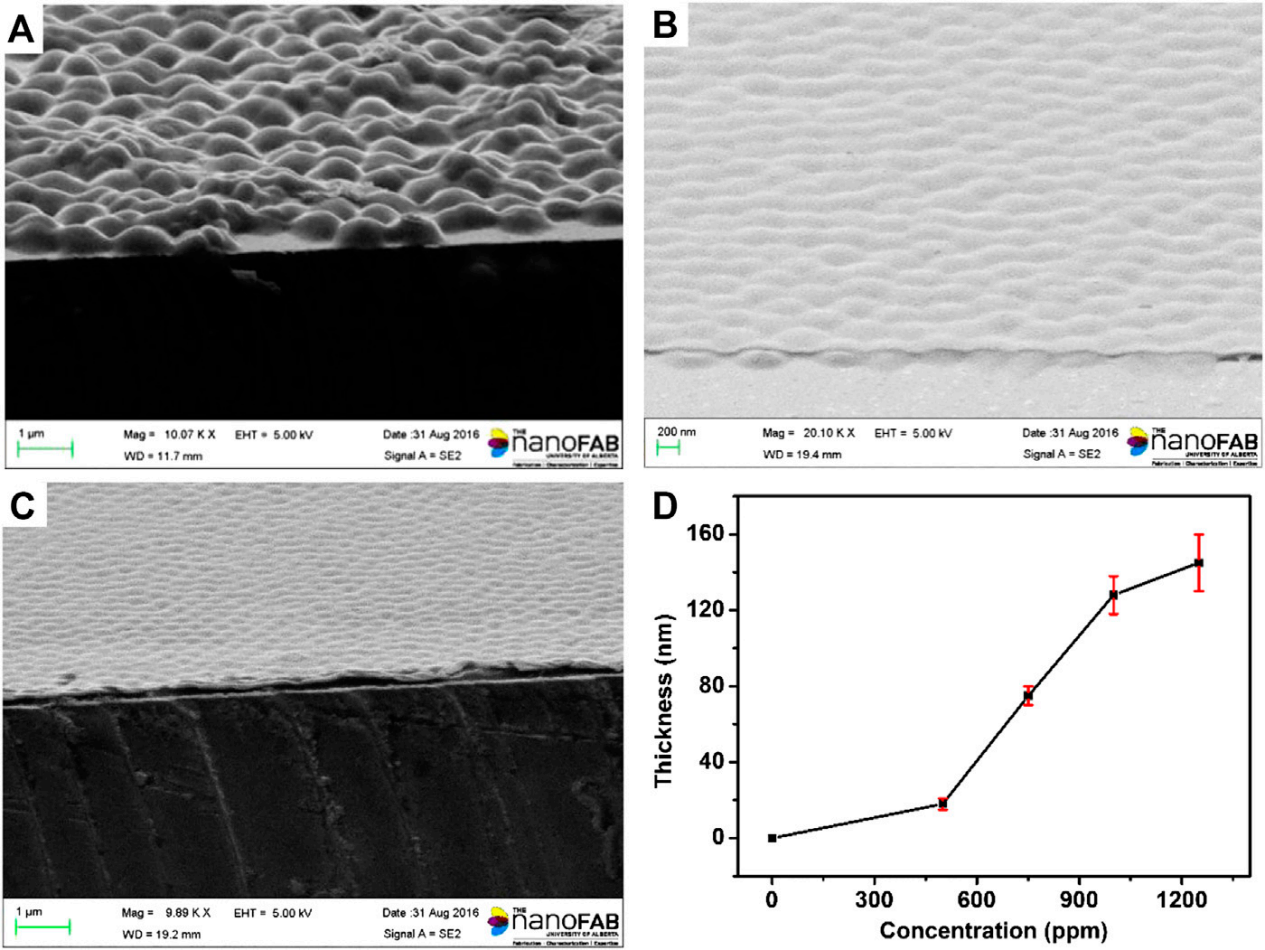
SEM images of microgels: **(A)** etalon without surface modification **(B)** etalon modified using 500 ppm dopamine **(C)** etalon modified using 1000 ppm dopamine **(D)** The thickness of polydopamine film as a function of dopamine concentration. These values are averages of three repeat analyses using three etalon devices.

The process of surface modification was monitored using X-Ray photoelectron spectroscopy (XPS) surface analysis technique, and the results were shown in Figure 3. The N1s XP spectra showed there is trace amount of N atoms on the surface of etalons. It dramatically increased when the etalon surface was modified using DTSP, which was attributed to the imide groups of DTSP. The intensity change of N atoms was also observed when tyrosinase was attached on the surface of etalons. As the polydopamine films were deposited on the surfaces of etalons, enormous intensity increase of N atoms in XPS spectrum was observed due to the high content of N atoms in polydopamine films. The S2p XP spectra also confirm the modification process on etalon surfaces (Figure 3B). The surface modification of etalon with tyrosinase caused the intensity change of S2p (162 eV) in XPS spectra. The peak of S2p at 162 eV almost disappeared, when polydopamine film was deposited on etalon surfaces. The phenomenon was due to the fact that the etalon surfaces and S atoms were covered by polydopamine films. The structure of polydopamine film on the etalon surface also has been investigated by Raman spectra, which has been shown in Figure 3C. The strong adsorption peak at 1650 cm^−1^ was assigned to the C = C stretching vibration of benzene. The polydopamine films were also investigated using SEM through observing the cross-section of etalons. In comparison of pure etalons, obvious polymeric films were found on the top of polydopamine modified etalons (Figure 4). The thickness of polydopamine films depends on the concentration of dopamine solution. As shown in Figure 4D, the thickness of polydopamine film increases from 18 to 128 nm as concentration increasing of dopamine from 500 ppm to 1250 ppm.

**FIGURE 5:**
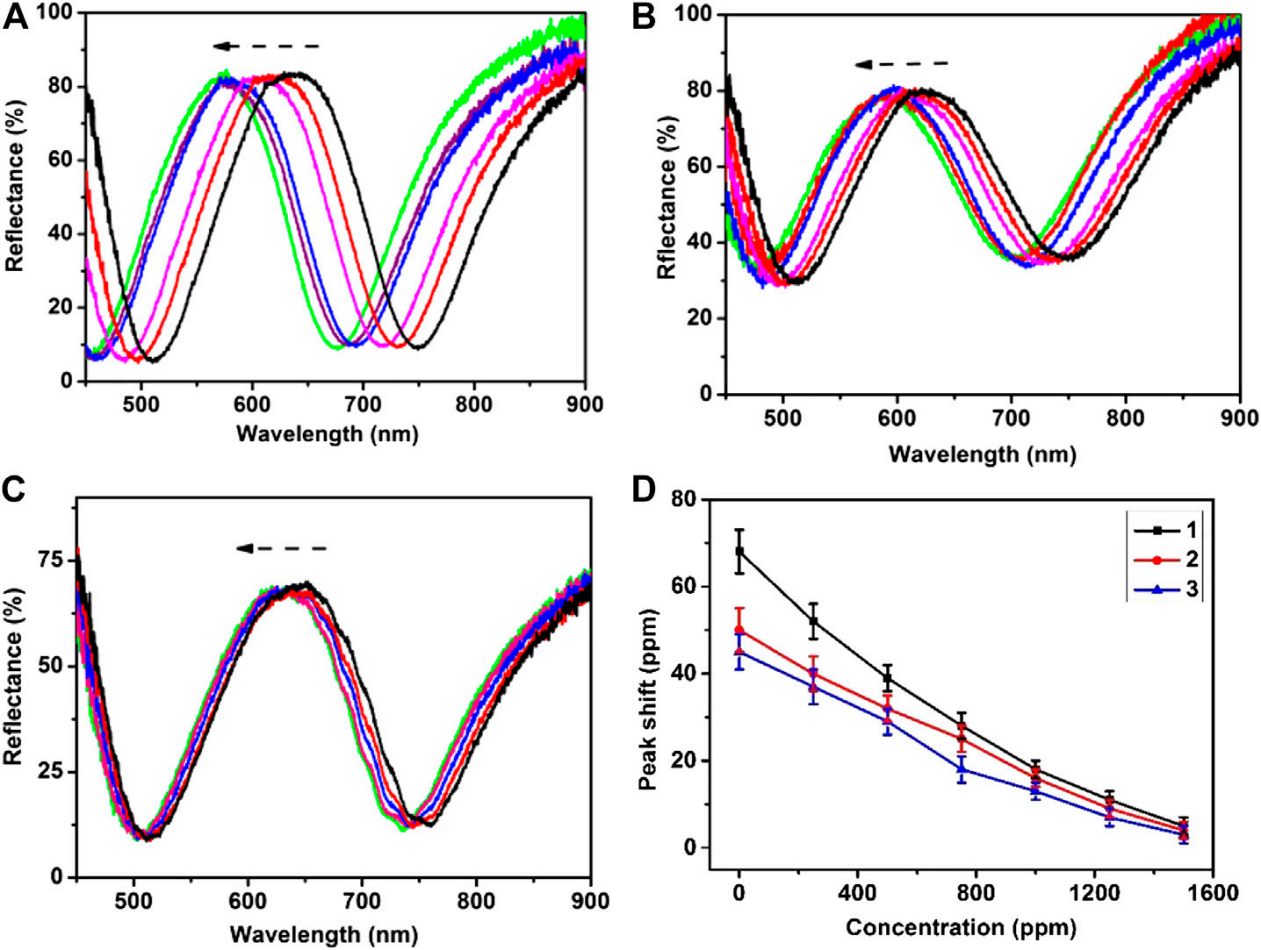
Reflectance spectra of etalon upon exposure to the ZnCl_2_ aqueous solution of 0.1 M recorded in 5 min: **(A)** etalon **(B)** etalon modified using 500 ppm dopamine **(C)** etalon modified using 1000 ppm dopamine **(D)** The peak shifts of reflectance spectra as a function of dopamine concentration: (1) in DI water, (2) in urine, (3) in serum. These values are averages of five repeat analyses using five etalon devices.

The response of the etalons to ZnCl_2_ was investigated at room temperature, and the reflectance spectra of etalons were shown in Figure 5. As can be seen, a characteristic peak at 630 nm was observed for pure etalon. When the etalon was exposed to 0.01M ZnCl_2_, the reflectance spectrum of etalon exhibited a blue-shift of 65nm and got stable around 5min. The blue-shift of reflectance spectrum was attributed to deswelling of microgels caused by ZnCl_2_. Our group has found in 2013 that the semi-transparent Au layer contains pores, which allow penetration of high molecular weight (Mw) polyelectrolytes (MW 100 000–200 000) from solution into etalons (Islam and Serpe, 2013). In the submission, ZnCl_2_ penetrates the semi-transparent Au layer and enters microgels. Zn^2+^ could coordinate with carboxylate groups of microgels with a ratio of 1: 2. In this case, Zn^2+^ acts like crosslinkers which make microgels shrink. Figure 5A demonstrates reflectance spectra of etalons subjecting to 0.01 M ZnCl_2_. When ZnCl_2_ present in the system, the reflectance spectra exhibit fast a blue-shift of 66 nm. The reflectance spectra become stable at 5 min. Therefore, all of spectrum data in the following experiment were read out at 5 min. The amplitudes of peak shift of reflectance spectra are inversely proportional to the concentration of dopamine used to surface modification of etalons. The etalons modified using 500 ppm dopamine exhibit a blue shift of 52 nm in response to 0.01 M ZnCl_2_, which is 14 nm less than that of pure etalon (66 nm). These values are averages of three repeat analyses using three different etalon devices. As the concentration of dopamine increases from 500 to 1250 ppm, the amplitude of spectra shift decreases from 52 to 10 nm. The similar trends of peak shifts were also observed when the detection was carried out in urine and serum. This phenomenon is attributed to thickness changes of dopamine films that block the diffusion of ZnCl_2_ through the film into microgels. The calibration curvesof spectral shift as a function of dopamine concentration were shown in Figure 5D. The sensor exhibited a limit of detection of 11.5 ppm in DI water, 18.7 ppm in urine, and 21.4 ppm in serum. Five devices were used to measure dopamine concentration at each point, which shows a maximum deviation of 8% between these devices. The result indicates a good device-to-device reproducibility for dopamine detection. Therefore, etalon can be used to quantitatively measure dopamine content in samples using the calibration curve. This method also can be used to detect other catecholamines with corresponding calibration curves.

## Conclusion

A method of polyphenols detection was developed based on “block” concept using etalon devices. First, the etalon surfaces were modified using tyrosinase that could oxidize polyphenols into polymeric films. The polymeric films impede the diffusion of ZnCl_2_ into etalons, which lead to different optical properties of etalons. The optical property of etalon is related to polyphenol concentration using surface modification of etalons. The concentration of polyphenol can be measured according to optical property change of etalons.

## Data Availability

The raw data supporting the conclusions of this article will be made available by the authors, without undue reservation.

## References

[B1] Afsari A. Sarraf M. J. (2020). Design of a hydrogen sulfide gas sensor based on a photonic crystal cavity using graphene. Superlattices Microstruct. 138, 106362. 10.1016/j.spmi.2019.106362

[B2] Alcalde B. Granados M. Saurina J. (2019). Exploring the antioxidant features of polyphenols by spectroscopic and electrochemical methods. Antioxidants 8, 523. 10.3390/antiox8110523 PMC6912615 31683530

[B3] Cai Z. Kwak D. H. Punihaole D. Hong Z. Velankar S. S. Liu X. (2015). A photonic crystal protein hydrogel sensor for candida albicans. Angew. Chem. Int. Ed. 54, 13036–13040. 10.1002/anie.201506205 26480336

[B4] Cai Z. Luck L. A. Punihaole D. Madura J. D. Asher S. A. (2016). Photonic crystal protein hydrogel sensor materials enabled by conformationally induced volume phase transition. Chem. Sci. 7, 4557–4562. 10.1039/c6sc00682e 30155102 PMC6016329

[B5] Cai Z. Sasmal A. Liu X. Asher S. A. (2017). Responsive photonic crystal carbohydrate hydrogel sensor materials for selective and sensitive lectin protein detection. ACS Sens. 2, 1474–1481. 10.1021/acssensors.7b00426 28934853

[B6] Desentis-Mendoza R. M. Hernández-Sánchez H. Moreno A. Rojas del C E. Chel-Guerrero L. Tamariz J. (2006). Enzymatic polymerization of phenolic compounds using laccase and tyrosinase from ustilago maydis. Biomacromolecules 7, 1845–1854. 10.1021/bm060159p 16768406

[B7] Espín J. C. Soler-Rivas C. Cantos E. Tomás-Barberán F. A. Wichers H. J. (2001). Synthesis of the antioxidant hydroxytyrosol using tyrosinase as biocatalyst. J. Agric. Food Chem. 49, 1187–1193. 10.1021/jf001258b 11312833

[B8] Fayeulle N. Meudec E. Boulet J. C. Vallverdu-Queralt A. Hue C. Boulanger R. (2019). Fast discrimination of chocolate quality based on average-mass-spectra fingerprints of cocoa polyphenols. J. Agric. Food Chem. 67, 2723–2731. 10.1021/acs.jafc.8b06456 30761902

[B9] Fu Y. Lin Y. Chen T. Wang L. (2012). Study on the polyfurfural film modified glassy carbon electrode and its application in polyphenols determination. J. Electroanal. Chem. 687, 25–29. 10.1016/j.jelechem.2012.09.040

[B10] Gao H. Yuan X. Wang Z. Gao Q. Yang J. (2020). Profiles and neuroprotective effects of Lycium ruthenicum polyphenols against oxidative stress-induced cytotoxicity in PC12 cells. J. Food Biochem. 44, e13112. 10.1111/jfbc.13112 31800113

[B11] Hashim S. N. N. S. Boysen R. I. Yang Y. Schwarz L. J. Danylec B. Hearn M. T. W. (2020). Parallel enrichment of polyphenols and phytosterols from Pinot noir grape seeds with molecularly imprinted polymers and analysis by capillary high-performance liquid chromatography electrospray ionisation tandem mass spectrometry. Talanta 208, 120397. 10.1016/j.talanta.2019.120397 31816764

[B12] Hou J. Li M. Song Y. (2018). Recent advances in colloidal photonic crystal sensors: materials, structures and analysis methods. Nano Today 22, 132–144. 10.1016/j.nantod.2018.08.008

[B13] Huang B. Wang Y. Mao C. (2019). Temperature-independent gas pressure sensor with high birefringence photonic crystal fiber-based reflective lyot filter. Sensors 19, 5312. 10.3390/s19235312 PMC6928900 31810370

[B14] Huang G. Li Y. Chen C. Yue Z. Zhai W. Li M. (2020). Hydrogen sulfide gas sensor based on titanium dioxide/amino-functionalized graphene quantum dots coated photonic crystal fiber. J. Phys. D: Appl. Phys. 53, 325102. 10.1088/1361-6463/ab89cc

[B15] Islam M. R. Serpe M. J. (2013). Penetration of polyelectrolytes into charged poly (N-isopropylacrylamide) microgel layers confined between two surfaces. Macromolecules 46, 1599–1606. 10.1021/ma302637n

[B16] Li Q. Zhou S. Zhang T. Zheng B. Tang H. (2020). Bioinspired sensor chip for detection of miRNA-21 based on photonic crystals assisted cyclic enzymatic amplification method. Biosens. Bioelectron. 150, 111866. 10.1016/j.bios.2019.111866 31744650

[B17] Liu R. Duan S. Bao L. Wu Z. Zhou J. Yu R. (2020). Photonic crystal enhanced gold-silver nanoclusters fluorescent sensor for Hg^2+^ ion. Analytica Chim. Acta 1114, 50–57. 10.1016/j.aca.2020.04.011 32359514

[B18] Massad-Ivanir N. Mirsky Y. Nahor A. Edrei E. Bonanno-Young L. M. Ben Dov N. (2014). Trap and track: designing self-reporting porous Si photonic crystals for rapid bacteria detection. Analyst 139, 3885–3894. 10.1039/c4an00364k 24930570

[B19] Matei A. O. Gatea F. Teodor E. D. Radu G. L. (2016). Polyphenols analysis from different medicinal plants extracts using capillary zone electrophoresis (CZE). Revista De Chim. 67, 1051–1055.

[B20] Nalewajko-Sieliwoniuk E. Malejko J. Pawlukiewicz A. Kojło A. (2016). A novel multicommuted flow method with nanocolloidal manganese (IV)-based chemiluminescence detection for the determination of the total polyphenol index. Food Anal. Methods 9, 991–1001. 10.1007/s12161-015-0274-8

[B21] Nalewajko-Sieliwoniuk E. Malejko J. Święczkowska M. Kowalewska A. (2015). A study on the selection of chemiluminescence system for the flow injection determination of the total polyphenol index of plant-derived foods. Food Chem. 176, 175–183. 10.1016/j.foodchem.2014.12.053 25624221

[B22] Ovchinnikov D. V. Bogolitsyn K. G. Druzhinina A. S. Kaplitsin P. A. Parshina A. E. Pikovskoi I. I. (2020). Study of polyphenol components in extracts of arctic brown algae of fucus vesiculosus type by liquid chromatography and mass-spectrometry. J. Anal. Chem. 75, 633–639. 10.1134/S1061934820050147

[B23] Parveen S. Memon S. Q. Siyal A. N. Memon N. Khuhawar M. Y. (2016). Large-volume sample staking of rice polyphenols prior to their determination by non-aqueous capillary electrophoresis. Food Anal. Methods 9, 2152–2160. 10.1007/s12161-015-0394-1

[B24] Ponder A. Hallmann E. (2019). The effects of organic and conventional farm management and harvest time on the polyphenol content in different raspberry cultivars. Food Chem. 301, 125295. 10.1016/j.foodchem.2019.125295 31387038

[B25] Ru Q. Xiong Q. Tian X. Chen L. Zhou M. Li Y. (2019). Tea polyphenols attenuate methamphetamine-induced neuronal damage in PC12 Cells by alleviating oxidative stress and promoting DNA repair. Front. Physiol. 10, 1450. 10.3389/fphys.2019.01450 31920684 PMC6915097

[B26] Solem E. Tuczek F. Decker H. (2016). Tyrosinase versus catechol oxidase: one asparagine makes the difference. Angew. Chem. Int. Ed. 55, 2884–2888. 10.1002/anie.201508534 26773413

[B28] Thangaraj R. Manjula N. Kumar A. S. (2012). Rapid simultaneous electrochemical sensing of tea polyphenols. Anal. Methods 4, 2922–2928. 10.1039/c2ay25563d

[B29] Xia X. Zhang X. Serpe M. J. Zhang Q. (2020). Microgel‐based devices as wearable capacitive electronic skins for monitoring cardiovascular risks. Adv. Mater. Technol. 5, 2070011. 10.1002/admt.201900818

[B30] Yasui M. Ishii T. (2014). A new method for detection of self-associated tea polyphenols using native-polyacrylamide gel electrophoresis. Bunseki Kagaku 63, 687–692. 10.2116/bunsekikagaku.63.687

[B31] Yuan B. Dinssa F. F. Simon J. E. Wu Q. (2020). Simultaneous quantification of polyphenols, glycoalkaloids and saponins in African nightshade leaves using ultra-high performance liquid chromatography tandem mass spectrometry with acid assisted hydrolysis and multivariate analysis. Food Chem. 312, 126030. 10.1016/j.foodchem.2019.126030 31911353

[B32] Zhang J.-T. Cai Z. Kwak D. H. Liu X. Asher S. A. (2014a). Two-dimensional photonic crystal sensors for visual detection of lectin concanavalin a. Anal. Chem. 86, 9036–9041. 10.1021/ac5015854 25162117

[B33] Zhang J.-T. Chao X. Liu X. Asher S. A. (2013). Two-dimensional array Debye ring diffraction protein recognition sensing. Chem. Commun. 49, 6337–6339. 10.1039/c3cc43396j 23743859

[B34] Zhang J.-T. Wang L. Luo J. Tikhonov A. Kornienko N. Asher S. A. (2011). 2-D array photonic crystal sensing motif. J. Am. Chem. Soc. 133, 9152–9155. 10.1021/ja201015c 21604702

[B35] Zhang Q.-M. Berg D. Mugo S. M. Serpe M. J. (2015b). Lipase-modified pH-responsive microgel-based optical device for triglyceride sensing. Chem. Commun. 51, 9726–9728. 10.1039/c5cc02853a 25983030

[B36] Zhang Q. M. Ahiabu A. Gao Y. Serpe M. J. (2015a). CO_2_-switchable poly (N-isopropylacrylamide) microgel-based etalons. J. Mater. Chem. C 3, 495–498. 10.1039/c4tc02600d

[B37] Zhang Q. M. Berg D. Duan J. Mugo S. M. Serpe M. J. (2016). Optical devices constructed from ferrocene-modified microgels for H_2_O_2_ sensing. ACS Appl. Mater. Inter. 8, 27264–27269. 10.1021/acsami.6b11462 27680293

[B38] Zhang Q. M. Wang W. Su Y.-Q. Hensen E. J. M. Serpe M. J. (2015c). Biological imaging and sensing with multiresponsive microgels. Chem. Mater. 28, 259–265. 10.1021/acs.chemmater.5b04028

[B39] Zhang Q. M. Xu W. Serpe M. J. (2014b). Optical devices constructed from multiresponsive microgels. Angew. Chem. Int. Ed. 53, 4827–4831. 10.1002/anie.201402641 24677793

[B40] Zhang R. Wang Q. Zheng X. (2018). Flexible mechanochromic photonic crystals: routes to visual sensors and their mechanical properties. J. Mater. Chem. C 6, 3182–3199. 10.1039/c8tc00202a

[B41] Zhang Y. Lan M. Lü J. P. Li J. F. Zhang K. Y. Zhi H. (2020). Antioxidant, anti-inflammatory and cytotoxic activities of polyphenols extracted from chroogomphus rutilus. Chem. Biodivers. 17, e1900479. 10.1002/cbdv.201900479 31667925

[B42] Zhao S. Chen C. Li Z. Yuan Z. Lu C. (2017). Hydroxyl radical induced chemiluminescence of hyperbranched polyethyleneimine protected silver nanoclusters and its application in tea polyphenols detection. Anal. Methods 9, 3114–3120. 10.1039/c7ay00903h

[B43] Zheng Q. Li W. Zhang H. Gao X. Tan S. (2020). Optimizing synchronous extraction and antioxidant activity evaluation of polyphenols and polysaccharides from Ya'an Tibetan tea (Camellia sinensis). Food Sci. Nutr. 8, 489–499. 10.1002/fsn3.1331 31993173 PMC6977498

